# Synthetic CT generation from CBCT and MRI using StarGAN in the Pelvic Region

**DOI:** 10.1186/s13014-025-02590-2

**Published:** 2025-02-04

**Authors:** Paritt Wongtrakool, Chanon Puttanawarut, Pimolpun Changkaew, Supakiet Piasanthia, Pareena Earwong, Nauljun Stansook, Suphalak Khachonkham

**Affiliations:** 1 Master of Science Program in Medical Physics, Department of Diagnostic and Therapeutic Radiology, Faculty of Medicine, Ramathibodi Hospital, Mahidol University, Bangkok, Thailand; 2 Department of Clinical Epidemiology and Biostatistics, Faculty of Medicine, Ramathibodi Hospital, Mahidol University, Bangkok, Thailand; 3 Faculty of Medicine, Chakri Naruebodindra Medical Institute, Ramathibodi Hospital, Mahidol University, Samut Prakan, Thailand; 4 Division of Radiation and Oncology, Department of Diagnostic and Therapeutic Radiology, Faculty of Medicine, Ramathibodi Hospital, Mahidol University, Bangkok, Thailand

**Keywords:** Radiotherapy, Synthetic CT, Deep learning, StarGAN, CycleGAN, CBCT, MRI

## Abstract

**Rationale and objectives:**

This study evaluated StarGAN, a deep learning model designed to generate synthetic computed tomography (sCT) images from magnetic resonance imaging (MRI) and cone-beam computed tomography (CBCT) data using a single model. The goal was to provide accurate Hounsfield unit (HU) data for dose calculation to enable MRI simulation and adaptive radiation therapy (ART) using CBCT or MRI. We also compared the performance and benefits of StarGAN to the commonly used CycleGAN.

**Materials and methods:**

StarGAN and CycleGAN were employed in this study. The dataset comprised 53 cases of pelvic cancer. Evaluation involved qualitative and quantitative analyses, focusing on synthetic image quality and dose distribution calculation.

**Results:**

For sCT generated from CBCT, StarGAN demonstrated superior anatomical preservation based on qualitative evaluation. Quantitatively, CycleGAN exhibited a lower mean absolute error (MAE) for the body (42.8 ± 4.3 HU) and bone (138.2 ± 20.3), whereas StarGAN produced a higher MAE for the body (50.8 ± 5.2 HU) and bone (153.4 ± 27.7 HU). Dosimetric evaluation showed a mean dose difference (DD) within 2% for the planning target volume (PTV) and body, with a gamma passing rate (GPR) > 90% under the 2%/2 mm criteria. For sCT generated from MRI, qualitative evaluation also favored the anatomical preservation provided by StarGAN. CycleGAN recorded a lower MAE (79.8 ± 14 HU for the body and 253.6 ± 30.9 HU for bone) compared with StarGAN (94.7 ± 7.4 HU for the body and 353.6 ± 34.9 HU for bone). Both models achieved a mean DD within 2% in the PTV and body, and GPR > 90%.

**Conclusion:**

While CycleGAN exhibited superior quantitative metrics, StarGAN was better in anatomical preservation, highlighting its potential for sCT generation in radiotherapy.

**Supplementary Information:**

The online version contains supplementary material available at 10.1186/s13014-025-02590-2.

## Introduction

Radiotherapy is a crucial component of cancer treatment, relying on precise radiation delivery to tumors while minimizing damage to healthy tissues. This balance is vital for maximizing tumor control probability and minimizing normal tissue complication probability. Achieving such precision involves several factors, one of which is accurate delineation of targets and organs at risk contours [1]. Traditionally, computed tomography (CT) simulation has served as the gold standard for contour delineation. However, magnetic resonance imaging (MRI) simulation offers soft-tissue visualization that is superior to CT, enhancing precision in contour delineation. Despite this advantage, MRI lacks Hounsfield units (HU), which are essential for determining electron density, a crucial factor for radiation dose calculation in radiotherapy planning [2]. Another important aspect of precision in radiation therapy is adaptive radiation therapy (ART). Traditional methods rely on a static snapshot of the patient’s anatomy from CT simulation, which may differ significantly from the patient’s anatomy on the day of treatment, leading to inaccurate radiation delivery. ART, on the other hand, adapts the treatment plan based on the daily patient anatomy acquired from daily imaging data using either MRI, if the center has an MR linear accelerator (Linac, Varian Medical Systems Inc., Palo Alto, CA, USA) or cone beam computed tomography (CBCT) for standard Linac [3, 4]. While both MRI and CBCT can be used for ART, each modality presents unique challenges. Despite its superior soft-tissue contrast, MRI can suffer from geometric distortions [3] and, as previously noted, lacks the necessary HU for accurate dose calculations. Conversely, although CBCT provides imaging data with HU, it is susceptible to insufficient image quality from various artifacts and the limited field of view (FOV) of the CBCT detector, compromising accurate dose calculation [4, 5].

Synthetic computed tomography (sCT) generation has been proposed as an effective solution to overcome these challenges. This process involves creating CT-like images using various techniques such as bulk density assignment, atlas-based methods, hybrid methods, and deep learning-based methods, with the latter proving to be the most effective [6]. sCT generation is a domain transfer task in deep learning, which can be implemented through various models [7]. Cycle generative adversarial networks (CycleGAN), a deep learning architecture based on GAN, is one of widely used models for sCT generation and has demonstrated effectiveness in numerous studies [7–17]. However, CycleGAN is limited to one-to-one translations, which restricts its versatility. In contrast, StarGAN [18–20] offers a more efficient alternative by enabling multi-domain translation (N-to-N translation) within a single model. By enabling the use of additional image domains, it potentially makes a model rely more on anatomical shape, thereby reducing the likelihood of anatomical distortions resulting from artificial intelligence (AI)-generated hallucinations. While StarGAN has not been used to generate sCT before in the field of radiology, it has already demonstrated the potential to perform multimodal MRI synthesis (T1, T1c, T2, and fluid-attenuated inversion recovery [FLAIR]) [21], cross-site MRI style transfer [22], and cross-machine/parameter CT style transfer [23].

In this study, we evaluated the performance of StarGAN in sCT generation using both CBCT and MRI in the pelvic region for qualitative and quantitative metrics, focusing on the quality of the synthetic images as well as the accuracy of dose calculations, which is a primary objective in radiation therapy. Additionally, we compared the performance of StarGAN with that of CycleGAN, a widely used model for sCT generation.

## Materials and methods

### Data acquisition

Following approval by the Institutional Review Board of Ramathibodi Hospital, Bangkok, Thailand (MURA2023/698), and in accordance with the Declaration of Helsinki, a retrospective study was conducted on 53 patients receiving radiotherapy. This cohort comprised 36 males and 17 females diagnosed with cancer at various sites, including the prostate (26 cases), rectum (13 cases), cervix (9 cases), bladder (2 cases), endometrium (1 case), pyriform (1 cases), and pancreas (1 cases). Among these patients, 18 had complete data for all three modalities, namely planning computed tomography (pCT), MRI, and CBCT; 15 only had data from pCT and MRI; and 20 cases only had data from pCT and CBCT. In total, the study comprised 53 pCT, 33 MRI, and 38 CBCT, as summarized in Table 1. All patients underwent treatment using the volumetric modulated arc therapy (VMAT) technique in the pelvic region. Patients with metallic prosthesis were excluded from this study.

**Table 1:** Overview of the number of cancer cases (images) included in the study

Data availability	Complete data	pCT and MRI only	pCT and CBCT only	Total
Prostate cancer	5 (220)	6 (264)	15 (660)	26 (1,144)
Rectum cancer	9 (396)	3 (132)	1 (44)	13 (572)
Cervical cancer	3 (132)	5 (220)	1 (44)	9 (396)
Bladder cancer	1 (44)	1 (44)	–	2 (88)
Endometrial cancer	–	–	1 (44)	1 (44)
Pyriform cancer	–	–	1 (44)	1 (44)
Pancreatic cancer	–	–	1 (44)	1 (44)
Total	18 (792)	15 (660)	20 (880)	53 (2,332)

The pCT images were acquired using the Optima 580 CT simulator (GE Healthcare, Milwaukee, WI, USA) prior to radiotherapy treatment. The acquisition parameters included a tube voltage of 120 kVp, a tube current of 50 mA, a slice thickness of 2.5 mm, dimensions of 512 × 512 pixels, and a pixel size of 1 × 1 mm^2^. The reconstruction algorithm was filtered back projection. These pCT images were utilized to delineate regions of interest (ROIs) and to create radiotherapy treatment plans using the Eclipse Treatment Planning System (TPS) version 16.1.0 (Varian Medical Systems Inc.). The pCT images, along with the corresponding treatment plans, served as a reference for sCT evaluation.

CBCT images were acquired during the first fraction of treatment using the On-Board Imager of the Linac (Varian Medical Systems Inc.) with a mounted half-fan bowtie filter. The acquisition parameters for CBCT included a tube voltage of 125 kVp, a tube current of 60 mA, a slice thickness of 2.0 mm, dimensions of 512 × 512 pixels, and a pixel size of 1 × 1 mm^2^. The Feldkamp–David–Kress (FDK) algorithm was used for reconstruction. This algorithm addresses the truncation artifact that occurs when tissue outside the FOV hardens the X-ray, leading to photon starvation, by extrapolating the projection data; adds zero-padding to address HU non-uniformities; and reduces streak artifacts before three-dimensional (3D) back-projection is performed [24].

MRI scans were acquired as soon as possible after CT simulation, with patient positioning and immobilization identical to what was used during CT simulation. A 1.5 T MR simulator (Magnetom Aera, Siemens, Erlangen, Germany) equipped with a flat tabletop (CIVCO Radiotherapy, Orange City, IA, USA) was used to acquire two-dimensional (2D) transverse T2-weighted turbo spin echo sequences. The MRI sequence covered a large FOV of 448 mm, encompassing all of the patient’s contours. The slice thickness was 2.5 mm with a 0 mm slice gap. Image resolution was 512 × 512 pixels, repetition time was 12,370.0 ms, echo time was 97 ms, and a high bandwidth of 200 Hz/pixel was used to reduce distortion. 3D distortion correction was also applied.

### Data preprocessing

Preprocessing began by performing rigid registration of the data using pCT as the reference. Following registration, slices that were not shared across all three modalities were excluded, resulting in 44 slices (images) in the center per patient. Then, the datasets were divided into three groups: training, validation, and testing. For the 18 patients with complete data, the dataset was divided into 9 for the training set, 5 for the validation set, and 4 for the test set. For the 15 patients with only pCT and MRI data, they were split into 14 for the training set and 1 for the test set. Finally, for the 20 patients with only pCT and CBCT data, they were divided into 19 for the training set and 1 for the test set.

**Fig. 1:**
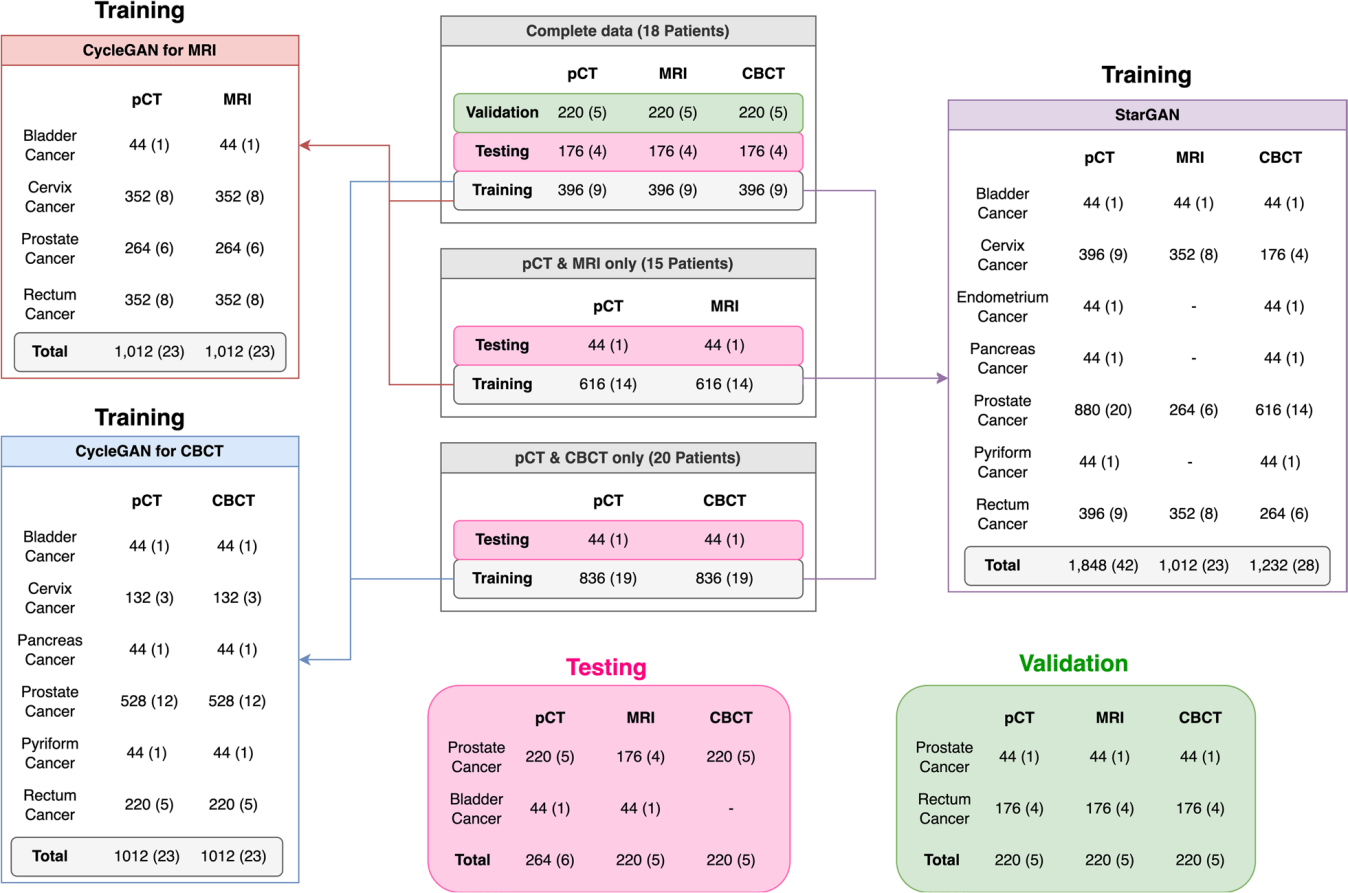
Overview of the sample distribution across the training, validation, and testing groups, as well as the datasets used for the development of the StarGAN and CycleGAN models

In the development of StarGAN, the model utilized data from 42 patients as a training set: 9 with complete data, 14 with MRI and pCT data, and 19 with pCT and CBCT data. To address the data imbalance in StarGAN, random oversampling was employed. For the development of CycleGAN for MRI, the training set consisted of 23 patients (9 with complete data and 14 with MRI and pCT data). In contrast, the CycleGAN for CBCT was developed using a training set of 23 patients (7 with complete data and 16 with pCT and CBCT data). An overview of the sample distribution across the different sets and models is illustrated in Fig. 1; additional details are presented in Table S1.

For CT and CBCT, the HU values were clipped into the range of [−1000, 1000] [9, 10, 13]. The treatment couch was removed using Otsu thresholding [25], max-connected component analysis, and followed by morphological opening. For MRI, the signal intensity was clipped to the range of [0, 1500] [12]. Finally, all data were normalized to a range of [−1, 1] [9, 10, 13] and resized to 512 × 512 pixels [9].

### Deep learning models and development

#### CycleGAN

CycleGAN consists of two primary components: generator (G) and discriminator (D). G is built upon an adapted version of the 2D UNet architecture [26], while D utilizes the PatchGAN [27] architecture. The loss function of D, denoted as ${L}_{D}$, is composed of two adversarial losses, formulated as follows:

$$L_{D} = mean\left( {\left( {1 - D_{A} \left( A \right) + D_{A} \left( {G_{A} \left( B \right)} \right)} \right)^{2} /2} \right) + mean\left( {\left( {1 - D_{B} \left( B \right) + D_{B} \left( {G_{B} \left( A \right)} \right)} \right)^{2} /2} \right).$$

The loss function of the generator ($L_{G}$) is composed of adversarial loss ($L_{adv,G}$), cycle consistency loss $(L_{cycle} )$ and identity loss $(L_{iden} )$. It is expressed as:

$$L_{G} = \lambda_{iden} L_{iden} + \lambda_{cycle} L_{cycle} + L_{adv,G} ,$$

where:

$$\begin{aligned} & L_{iden} = mean\left( {\left| {A - G_{A} \left( A \right)} \right|} \right) + mean\left( {\left| {B - G_{B} \left( B \right)} \right|} \right); \\ & L_{cycle} = mean\left( {\left| {A - G_{A} \left( {G_{B} \left( A \right)} \right)} \right|} \right) + {\text{mean}}\left( {\left| {B - G_{B} \left( {G_{A} \left( B \right)} \right)} \right|} \right);\;and \\ & L_{adv,G} = mean\left( {\left( {D_{A} \left( {G_{A} \left( B \right)} \right) - 1} \right)^{2} } \right) + mean\left( {\left( {D_{B} \left( {G_{B} \left( A \right)} \right) - 1} \right)^{2} } \right). \\ \end{aligned}$$

In these equations, $G_{A}$​ and $G_{B}$​ ​ are the generators for translating images from domain A to domain B and vice versa. A and B are images from domain A and domain B, respectively.

#### StarGAN

StarGAN utilizes the SuperStarGAN structure [20] and the 2D UNet architecture (https://github.com/KKhyeok/SuperstarGAN). StarGAN is composed of three components: generator (G), discriminator (D, and classifier (C). D uses the loss function ($L_{D}$) that incorporates two adversarial losses derived from real and synthetic images, expressed as follows:

$$L_{D} = mean\left( {ReLU\left( {1 + \left( {D\left( {G\left( {x, label_{target} } \right)} \right)} \right)} \right)} \right) + mean\left( {ReLU\left( {1 - D\left( x \right)} \right)} \right)$$.

The loss function of G ($L_{G}$) is composed of classification loss (L_cls_), reconstruction loss (L_rec_), and adversarial loss (L_adv_). G takes two inputs, $x$ and $label_{T}$, to translate input $x$ to a given domain $T$. The loss function for the G is defined as:

$$L_{G} = \lambda_{cls} L_{cls} + \lambda_{rec} L_{rec} + L_{adv} ,$$

where:

$$\begin{aligned} & L_{cls} = CrossEntropy\left( {C\left( {G(X,label_{target} } \right), label_{target} } \right); \\ & L_{rec} = mean\left( {\left| {X - G\left( {\left( {G(X,label_{target} } \right),label_{org} } \right)} \right|} \right);\;and \\ & L_{adv} = - mean\left( {D\left( {G\left( {X,label_{target} } \right)} \right)} \right). \\ \end{aligned}$$

Lastly, the loss function of C (L_c_) consists solely of the classification loss for the real input:

$$L_{c} = CrossEntropy\left( {C\left( X \right),label_{org} } \right).$$

In this study, the hyperparameter $\lambda_{c}$ and $\lambda_{rec}$ were set to 0.25 and 1.3, respectively, as defined in the original SuperStarGAN paper [20].

### Model development

For both CycleGAN models, the hyperparameters were as follows: a batch size of 4 and a learning rate of 0.0001. The Adam optimizer [28] was employed. Training was conducted for a total of 500 epochs, with the learning rate decaying after 200 epochs. In this process, the learning rate was reduced by a factor calculated as the initial learning rate divided by the number of decaying epochs. Consequently, the learning rate gradually approached zero in the later epochs and became 0 after the last epoch.

In contrast, the development of the StarGAN model utilized a transfer learning approach. First, the model underwent a pre-training phase using only data from the pCT and MRI datasets for 250 epochs. Then, the weights obtained from the model at epoch 250 served as the initial weights for transfer learning. During the transfer learning phase, the model was trained with all datasets, including pCT, MRI, and CBCT, for an additional 250 epochs. The hyperparameters for the pre-training and transfer learning phases were the same as those used in the CycleGAN training, except for the number of decaying epochs during the pre-training phase, which was set to 0. All models were developed using the PyTorch framework [29] on a NVIDIA Tesla V100S PCIe 32 GB GPU. The code used in this study can be accessed at https://github.com/Paritt/sCT-via-StarGAN-and-CycleGAN.git.

### Evaluation

After completing training, generator of both CycleGAN and StarGAN from the best-performing epoch (based on minimum mean absolute error in validation dataset) was selected to generate sCT from 5 CBCT and 5 MRI in the testing dataset. Additionally, after generating sCT images, they were converted back to HU values in the [−1000,1000] range for qualitative and quantitative evaluation.

#### Qualitative evaluation

For qualitative evaluation, visual examination was conducted to assess the overall image quality and anatomical preservation of the generated sCT. Additionally, HU difference maps were generated by subtracting the HU values of the sCT and/or CBCT images from their corresponding pCT images on a pixel-by-pixel basis, allowing for visualization of the difference between the images. Furthermore, histograms of the images were plotted and compared among all image types, providing more insight into the overall HU differences.

#### Quantitative evaluation

Quantitative evaluation comprised three aspects: image similarity, geometric accuracy, and dosimetric accuracy. These quantitative metrics were utilized for statistical comparison using the Wilcoxon signed-rank test [30] between sCT and CBCT, as well as between sCT generated by CycleGAN and sCT generated by StarGAN.

For the entire image, the comparison involved calculating the structural similarity index (SSIM) and the peak signal-to-noise ratio (PSNR) between the generated sCT and the reference pCT images. SSIM is defined as:

$$SSIM = \frac{{\left( {2\mu_{sCT} \mu_{pCT} + c_{1} } \right)\left( {2\sigma_{sCT,pCT} + c_{2} } \right)}}{{\left( {\mu_{sCT}^{2} + \mu_{pCT}^{2} + c_{1} } \right)\left( {\sigma_{sCT}^{2} + \sigma_{pCT}^{2} + c_{2} } \right)}},$$

where $\mu_{sCT}$ and $\mu_{pCT}$ are the average HU of sCT and pCT, respectively; $\sigma_{sCT}$ and $\sigma_{pCT}$ are the variance of sCT and pCT, respectively; and $\sigma_{sCT,pCT}$ is the covariance of sCT and pCT. Noted that $c_{1}$ is $\left( {0.01L} \right)^{2}$ and $c_{2}$ is $\left( {0.03L} \right)^{2}$, where *L* is the range of HU in the pCT image.

PSNR is defined as:

$${\text{PSNR}} = 10 \cdot {\text{log}}_{10} \left( {\frac{{R^{2} }}{{{\text{MSE}}}}} \right),$$

where R is the maximum possible pixel value of the image (1000 in this case), and MSE is the mean squared error between the sCT and the pCT image, calculated as the mean of $\left( {{\text{sCT}}\left( i \right) - {\text{pCT}}\left( i \right)} \right)^{2}$. Here, sCT(i) and pCT(i) denote the HU of voxel i.

Furthermore, ROIs including bone and body were segmented automatically from the sCT and pCT images using a thresholding method. Bone was defined as HU > 250, and the body was defined as > −200 HU. These ROIs were utilized for geometric accuracy assessment, evaluated using the Dice similarity coefficient (DSC), calculated as follows:

$${\text{DSC}} = 2\left( {\frac{{{\text{ROI}}_{{{\text{sCT}}}} \cap {\text{ROI}}_{{{\text{pCT}}}} }}{{{\text{ROI}}_{{{\text{sCT}}}} + {\text{ROI}}_{{{\text{pCT}}}} }}} \right).$$

The tolerance criterion of a DSC > 0.8 [31], commonly used in image registration, was adopted to determine acceptable geometric accuracy since there are no standard criteria for sCT evaluation. Furthermore, the mean absolute error (MAE) for each ROI in the pCT, containing M voxels, was computed using the formula:

$${\text{MAE}} = \frac{1}{{{\text{M}}\mathop \sum \nolimits_{{{\text{i}} \in {\text{ROI}}_{{{\text{pCT}}}} }} \left| {{\text{sCT}}\left( {\text{i}} \right) - {\text{pCT}}\left( {\text{i}} \right)} \right|}}.$$

The generated sCT images were imported into the Eclipse TPS to assess dosimetric accuracy. To evaluate dosimetric similarity, the sCT treatment plan was set identical to the treatment plan of its corresponding pCT image by copying the VMAT treatment plan from pCT to it corresponding sCT, and the dose was recalculated using the analytical anisotropic algorithm (AAA) with a grid size of 2.5 × 2.5 mm^2^. The dose difference (DD) between pCT and sCT at a specific point of the dose–volume Histogram (DVH), including planning target volume (PTV) D95%, PTV Dmean, PTV D2%, Body D2%, and Body Dmean, were computed with the following equation:

$${\text{DD}} = 100\left( {\frac{{Dose_{sCT} - Dose_{pCT} }}{{Dose_{pCT} }}} \right).$$

The DD tolerance criterion was set as 2%, which was defined in previous studies [32, 33].

Additionally, gamma analysis was conducted using a fast algorithm designed for gamma evaluation in 3D [34]. The gamma analysis criteria were set at 2% DD and 2 mm distance-to-agreement (DTA) with global normalization and a low-dose cutoff of 10% and 50% were applied.

## Results

Both CycleGAN models (CBCT and MRI) trained for 500 epochs, with CBCT taking 3 days, 5 min, and 46 s (lowest MAE: 54.32 HU at epoch 78) and MRI taking 3 days, 59 min, and 24 s (lowest MAE: 77.78 HU at epoch 369). The combined training time was 6 days, 1 h, 5 min, and 10 s. StarGAN pre-trained for 250 epochs in 2 days, 12 h, 53 min, and 45 s, then transfer learned for another 250 epochs in 3 days, 19 h, 1 min, and 40 s (a total of 5 days, 7 h, 55 min, and 25 s). For StarGAN, the lowest combined MAE (MAE of CBCT + MAE of MRI) was 166.05 HU at epoch 259 (CBCT: 68.30 HU; MRI: 97.76 HU). It is important to note that all quantitative results showed no significant differences (p > 0.05) between sCT and CBCT, as well as between sCT generated by CycleGAN and sCT generated by StarGAN.

### Qualitative evaluation

**Fig. 2:**
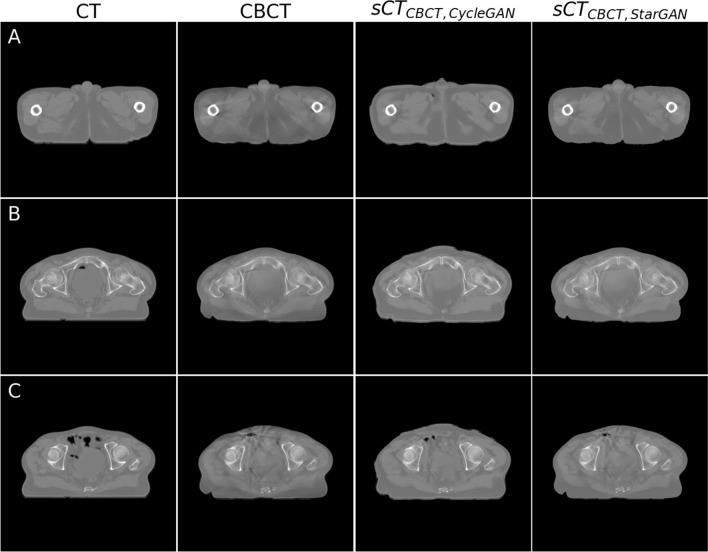
Examples of sCT generated from CBCT using CycleGAN and StarGAN in the testing dataset. **A** sCT reduced streak artifacts from bone in CBCT, although CycleGAN introduced unreal air regions. **B** sCT mitigated ring and cupping artifacts in CBCT. **C** sCT reduced star artifacts originating from air regions in CBCT

**Fig. 3:**
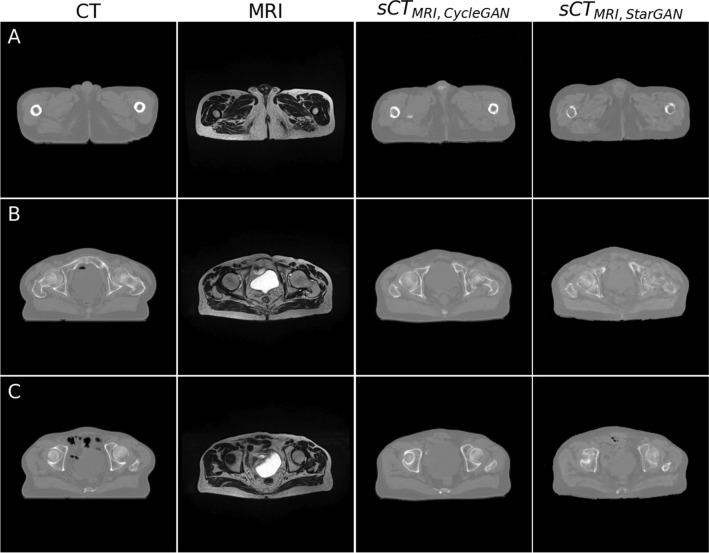
Examples of sCT generated from MRI using CycleGAN and StarGAN in the test dataset. **A** CycleGAN produced sharper images compared with StarGAN. **B** and **C** CycleGAN generated a more distorted bladder shape compared with the original MRI, while StarGAN preserved the shape more accurately

The visual examination of sCT generated from CBCT and MRI data in the testing dataset is illustrated in Figs. 2 and 3, respectively. For sCT generated from CBCT, CycleGAN effectively reduced streak artifacts from bone (Fig. 2A), ring and cupping artifacts (Fig. 2B), and star artifacts from air (Fig. 2C), while maintaining anatomical accuracy, albeit with some impact on air and bladder regions affected by star and cupping artifacts. In contrast, although StarGAN showed poorer artifact reduction, it produced sharper image and exceled in preserving anatomical accuracy, as evidenced by its ability to maintain air and bladder structures (Fig. 2B, C). Moreover, sCT obtained with StarGAN did not have an unreal air region like sCT obtained with CycleGAN (Fig. 2A).

For sCT derived from MRI, compared with StarGAN, CycleGAN displayed a drawback in maintaining anatomy, as illustrated by the distorted bladder shape in Fig. 3B and C, which deviates from the original MRI. However, sCT obtained with CycleGAN displayed a sharper image.

**Fig. 4:**
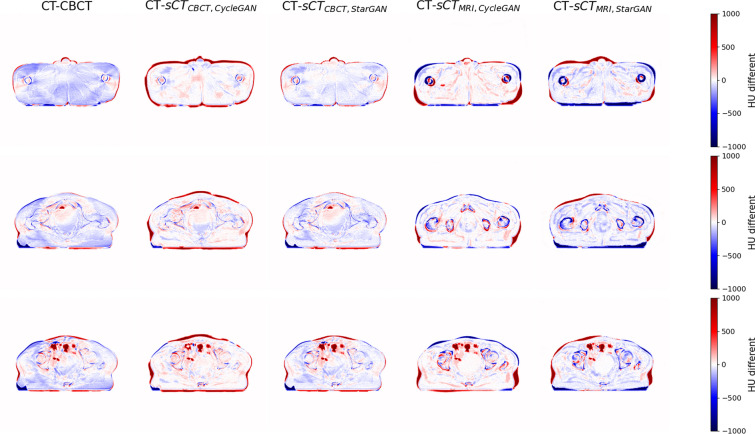
HU difference maps of sCT and pCT. The red color indicates a higher HU value than pCT, and the blue color indicates a lower HU value than pCT. CBCT had a lower HU values compared with pCT, while sCT had HU values closer to pCT

Figure 4 illustrates the HU difference map between pCT, CBCT, and sCT images. In the case of sCT generated from CBCT, CycleGAN outperformed StarGAN in improving HU values, as evidenced by a greater presence of white colors in the HU difference map. StarGAN also presented superior improvement in HU values for sCT generated from MRI. It is noteworthy that the map edges showed significant differences due to the differences in anatomies and/or patient positioning of the original CBCT, MRI, and pCT.

**Fig. 5:**
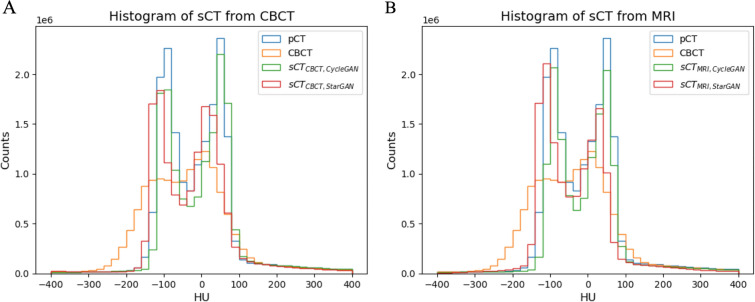
Histograms of HU values for sCT derived from (**A**) CBCT and **B** MRI in the body region

Figure 5 further confirms the improvement in the HU values of sCT: The histograms show a distribution of HU values in the body region of sCT that more closely resembles the distribution observed in pCT, as opposed to the distribution in CBCT. Furthermore, the histogram of sCT by StarGAN better resembles the distribution of pCT compared with the histogram of sCT by CycleGAN. This holds true for both sCT generated from CBCT and MRI. It is noteworthy that the histogram of sCT generated from MRI more closely resembles the distribution of pCT than that of CBCT. This indicates that, even when derived from MRI, sCT shows greater similarity to pCT.

The histograms illustrate a closer distribution for sCT and pCT compared with CBCT and pCT.

### Quantitative evaluation

The SSIM and PSNR results are shown in Additional file 1: Table S2. The SSIM was slightly higher for sCT derived from CBCT than the SSIM for CBCT (0.88 ± 0.02 for both CycleGAN and StarGAN vs. 0.86 ± 0.03 for CBCT), while the SSIM of sCT derived from MRI was slightly lower than the SSIM for CBCT (0.85 ± 0.03 and 0.82 ± 0.02 for CycleGAN and StarGAN, respectively). On the other hand, the PSNR was slightly higher for CBCT than the PSNR for sCT derived from CBCT (25.73 ± 0.69 and 29.18 ± 1.07 for CycleGAN and StarGAN, respectively), and the PSNR was notably higher for sCT derived from MRI (24.74 ± 0.89 and 23.81 ± 0.89 for CycleGAN and StarGAN, respectively).

When comparing StarGAN with CycleGAN, in terms of sCT derived from CBCT both models achieved very similar values, with StarGAN yielding a slightly higher PSNR. For sCT derived from MRI, CycleGAN had a slightly higher SSIM and PSNR. Importantly, there were no significant differences in the SSIM and PSNR, indicating no significant difference between sCT and CBCT, even for sCT derived from MRI.

The MAE results are shown in Table 2. StarGAN consistently yielded a higher MAE than CycleGAN. When compared with CBCT, sCT generated from CBCT showed a lower MAE for the body and bone regions, whereas sCT generated from MRI exhibited a higher MAE for both body and bone. Although these differences were not statistically significant, they suggest trends in the data.

**Table 2:** Mean MAE ± SD of body and bone of CBCT and sCT derived from CBCT and MRI (obtained with StarGAN and CycleGAN) using pCT as a reference in testing dataset

ROI	Base image	Model	MAE ± SD	p-value
Body	CBCT	CBCT	65.67 ± 11.26	–
		CycleGAN	42.77 ± 4.28	0.0625
		StarGAN	50.81 ± 5.16	
	MRI	CycleGAN	79.77 ± 13.96	0.1250
		StarGAN	94.65 ± 7.41	
Bone	CBCT	CBCT	135.94 ± 25.36	–
		CycleGAN	138.17 ± 20.29	0.0625
		StarGAN	153.36 ± 27.67	
	MRI	CycleGAN	253.62 ± 30.85	0.0625
		StarGAN	353.58 ± 34.85	

Table 3 displays the DSC values. All CBCT and sCT images achieved a DSC that exceeded the 0.8 threshold for the body region. Considering the sCT and CBCT comparison, for the body region, sCT and CBCT demonstrated a similar DSC. For the bone region, sCT derived from CBCT showed small differences compared with CBCT. However, sCT generated from MRI exhibited more notable differences. Additionally, when comparing sCT generated from CBCT using StarGAN to that produced by CycleGAN, both methods yielded a similar DSC. Notably, the DSC for CycleGAN (0.57 ± 0.10) was higher than that for StarGAN (0.38 ± 0.12) in the context of sCT generated from MRI.

**Table 3:** Mean DSC ± SD for CBCT and sCT derived from CBCT and MRI (obtained with StarGAN and CycleGAN) using pCT as a reference in the testing dataset

ROI	Base image	Model	DSC ± SD	p-value	Criteria [31]
Body	CBCT	CBCT	0.97 ± 0.02	–	> 0.8
		CycleGAN	0.98 ± 0.01	0.0588	
		StarGAN	0.99 ± 0.00		
	MRI	CycleGAN	0.97 ± 0.01	0.1573	
		StarGAN	0.96 ± 0.01		
Bone	CBCT	CBCT	0.80 ± 0.05	–	
		CycleGAN	0.80 ± 0.05	0.0679	
		StarGAN	0.77 ± 0.06		
	MRI	CycleGAN	0.57 ± 0.10	0.0625	
		StarGAN	0.38 ± 0.12		

**Fig. 6:**
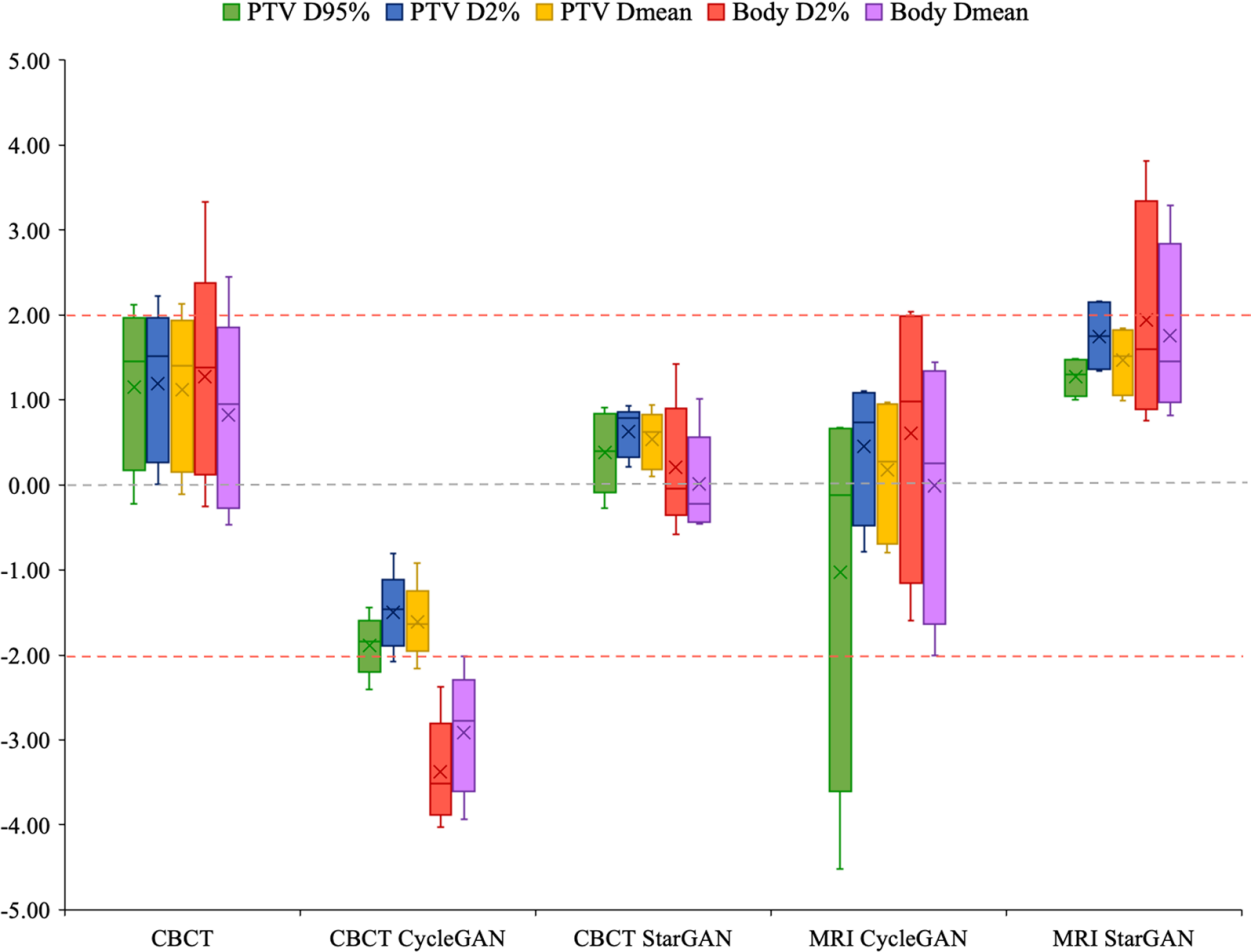
A boxplot of the DVH DD of CBCT and sCT derived from CBCT and MRI obtained with StarGAN and CycleGAN

The DVH DD between CBCT and sCT, compared with the reference pCT, is illustrated in Fig. 6, with detailed values provided in Additional file 1: Table S3. Overall, for both CBCT and sCT, most values fell within or close to the 2% threshold, indicating acceptable dose accuracy. However, for sCT derived from CBCT using CycleGAN, body Dmean and D2% slightly exceeded the 2% threshold, although this difference was not statistically significant compared with CBCT. Moreover, sCT derived from CBCT using StarGAN demonstrated dose differences closer to 0% than CycleGAN across all ROIs (Fig. 6).

When comparing CycleGAN to StarGAN for sCT derived from CBCT, StarGAN showed consistently smaller dose differences, with values closer to 0%, indicating better dose accuracy with minimal deviations. Conversely, for sCT derived from MRI, CycleGAN achieved dose differences closer to 0%, although with greater variability compared with StarGAN.

These results suggest that sCT derived from CBCT can effectively reduce dose differences compared with CBCT, which could be beneficial for ART by enabling more accurate dose calculations. StarGAN appears to outperform CycleGAN for sCT derived from CBCT by reducing dose deviations. For sCT derived from MRI, the fact that most dose differences fall within or near the 2% threshold indicates that MRI-based workflows, supported by sCT generation, can provide accurate dose calculations. In this context, StarGAN performs comparably to CycleGAN.

**Fig. 7:**
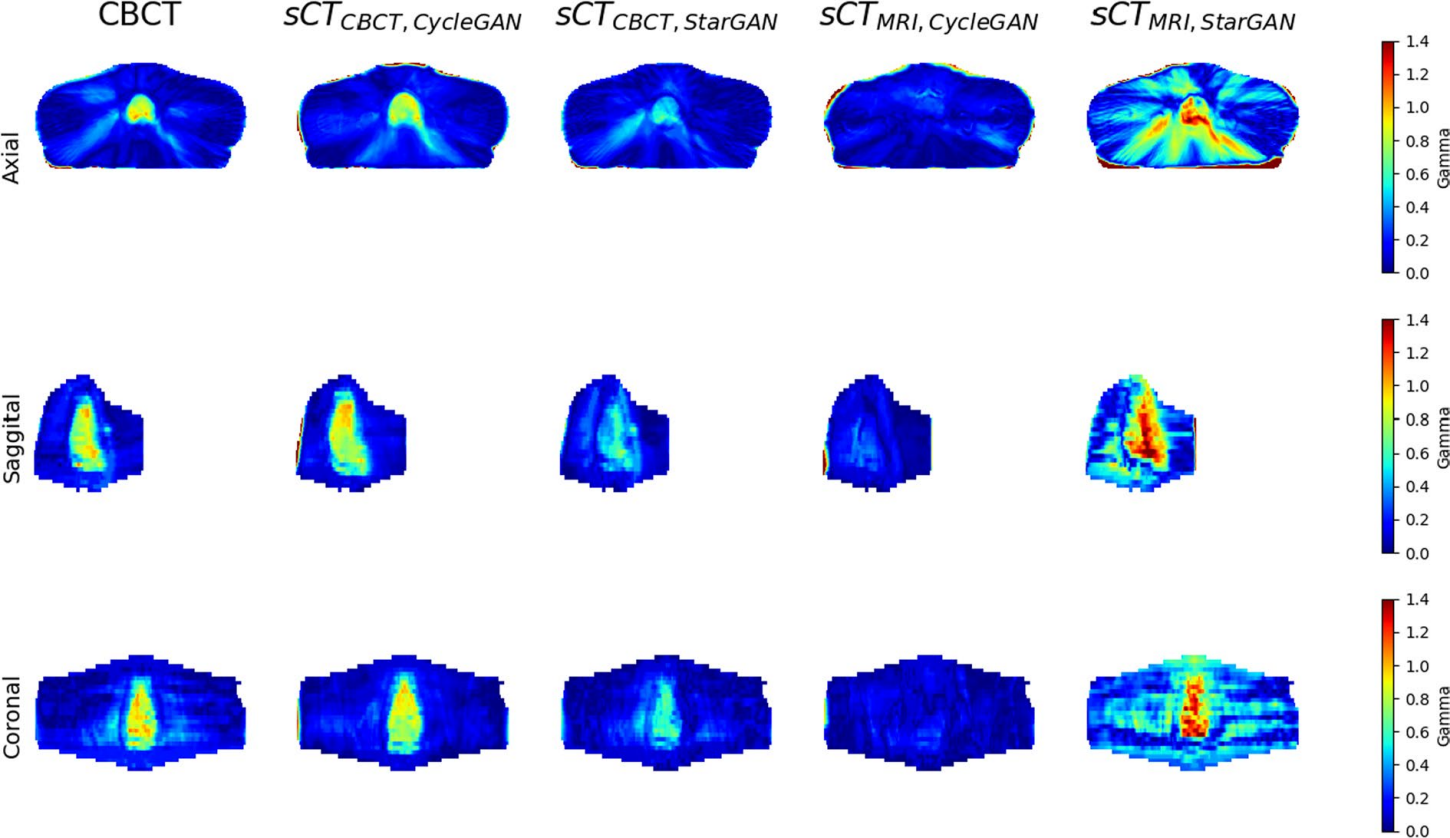
Example gamma maps of CBCT and all sCT images were calculated using the 2%/2 mm criteria and global normalization at a low-dose cutoff of 10%

Table 4 summarizes the percentage gamma passing rate (%GPR) calculated using global normalization with a low-dose cutoff of 10% (additional data using a 50% cutoff are provided in Additional file 1: Table S4). The table also includes p-values derived from the Wilcoxon signed-rank test, comparing each result to the CBCT for its respective gamma criterion. %GPR was > 90% for all models, including CBCT. There are no statistically significant differences between CBCT and each sCT. The example of gamma maps using 2%/2 mm criteria and global normalization with a low-dose cutoff of 10% is presented in Fig. 7.

**Table 4:** Mean %GPR) ± SD using global normalization in the testing dataset at a low-dose cutoff of 10%

Gamma criteria	Base image	Model	%GPR ± SD	p-value
2%/2 mm	CBCT	CBCT	99.64 ± 0.30	–
		CycleGAN	98.41 ± 0.83	0.0625
		StarGAN	99.79 ± 0.15	0.1250
	MRI	CycleGAN	96.18 ± 3.48	0.3125
		StarGAN	94.49 ± 2.78	0.0625

## Discussion

We investigated sCT generation using StarGAN, a model capable of generating sCT from both CBCT and MRI using a single model. We evaluated the performance of StarGAN compared with CycleGAN, a widely used model, in the pelvic region to identify the strengths and limitations of each model in terms of both qualitative and quantitative performance.

### sCT derived from CBCT

For sCT derived from CBCT, based on visual inspection, StarGAN performed better than CycleGAN, as evident by sharper image and ability to preserve anatomy while reducing artifacts (Fig. 2). This outcome is likely because StarGAN learns from the HU values in CBCT and the signal intensity in MRI; hence, it is more reliant on overall anatomical features rather than HU values or signal intensity alone. Nevertheless, both StarGAN and CycleGAN reduced artifacts and improved image quality, findings consistent with previous studies [8–10, 13]. Both models also maintained most of the anatomical features, but the most challenging areas were air and the bladder region. This finding is similar to the study by Eckl et al. [10]. It is likely due to the fact that air and the bladder varied in shape across the dataset, making them harder to learn.

In terms of quantitative analysis, the MAE for the body region was 42.77 ± 4.28 HU (CycleGAN) and 50.81 ± 5.16 HU (StarGAN). These values fall within the range of previous studies that employed GAN-based methods (8.1–97.4 HU) [8–10, 35–40]. The MAE for the bone region was 138.17 ± 20.29 HU (CycleGAN) and 153.36 ± 27.67 HU (StarGAN), higher compared with 118 ± 25.9 HU reported by Eckl et al. [10]. The difference is likely due to the fact that Eckl et al. [10] used an expert physician to manually delineate the ROIs and applied a negative margin to compensate for anatomical differences. Moreover, our training data had greater heterogeneity compared with Eckl et al. [10], who focused exclusively on prostate and seminal vesical cancer. Additionally, Eckl et al. [10] utilized ADMIRE (ADvanced Medical Image Registration Engine), commercial software from Elekta that applies a 2D CycleGAN. The modeling details and training approaches used by this commercial software are not publicly available, which limits the reproducibility of their results.

In terms of dosimetric accuracy, StarGAN showed superior performance compared with CycleGAN in reducing DD in the PTV and body to meet the 2% criterion with only minor deviations. Moreover, %GPR was similar for both models, although StarGAN performed slightly better. This may be because %GPR was already high in CBCT. We achieved a similar %GPR compared with what Eckl et.al. [10] reported: 98.5% ± 1.7% for the 2%/2 mm criteria. When using a low-dose cutoff of 50% and the 2%/2 mm criteria, %GPR was 97.65% ± 3.53% for CycleGAN and 100.0% ± 0.0% for StarGAN.

### sCT derived from MRI

For sCT derived from MRI, visual examination indicated that both CycleGAN and StarGAN can generate sCT from MRI that resembles CT, a finding similar to other studies [12, 16]. sCT from CycleGAN appeared sharper than sCT from StarGAN, but similarly to sCT derived from CBCT, CycleGAN struggled to preserve anatomy, as the bladder in sCT using CycleGAN was misshapen compared with the original MRI (Fig. 3B and C). This finding is similar to the study by Brou Boni et al. [12] and Liu et al. [15]. This phenomenon is commonly known as AI hallucination, and it also happened with StarGAN, which introduced unreal air pockets to sCT (Fig. 3C). However, StarGAN still generated HU values close to pCT, evident in the HU difference map and histogram, though not as effectively as CycleGAN.

Both models tended to have a higher MAE than CBCT and sCT derived from CBCT. This outcome is because generating sCT from MRI is more challenging than generated sCT from CBCT, as MRI differs more significantly from pCT than CBCT. Compared with the existing literature, our body MAE of 79.77 ± 13.96 HU for CycleGAN and 94.65 ± 7.41 HU for StarGAN are higher than the range reported in prior GAN-based methods (29.5–65.0 HU) [12, 41–45]. Several factors may account for this discrepancy. First, we utilized images with a resolution of 512 × 512 pixels, which corresponds to a clinical image size. These larger images pose a more challenging optimization task compared with the lower-resolution images often used in other studies. Second, the greater heterogeneity in our training set may have contributed to the observed differences.

In terms of dosimetric accuracy, CycleGAN could reduce DD in the PTV and body more than StarGAN, but with a high deviation. Additionally, both StarGAN and CycleGAN DD in PTV and body fell under or near 2% criterion indicate its usability. Moreover, the %GPR for both models was consistently high, exceeding 90% under the 2%/2 mm criteria, comparable to previous studies [12, 41] with minor differences due to variations in image resolution and data heterogeneity, as discussed previously.

### StarGAN vs CycleGAN

Overall, StarGAN tended to exhibit superior performance compared with CycleGAN for sCT derived from CBCT but inferior performance for sCT derived from MRI. However, StarGAN demonstrated superior preservation of anatomical features (based on visual inspection) in both domains. This difference in performance is possibly due to the complexity of the task undertaken by StarGAN. Unlike CycleGAN, which primarily focuses on the transformation from one domain to another, StarGAN is tasked with optimizing models for multiple objectives. Specifically, StarGAN must facilitate transformations not only from CBCT to pCT or MRI to pCT, but also including CBCT to MRI, MRI to CBCT, pCT to CBCT, and pCT to MRI. Consequently, this multidimensional optimization presents more challenges for the model [23]. This situation is similar to previous studies that have compared StarGAN and CycleGAN in different applications: CycleGAN demonstrated superior performance in generative tasks [23, 46–48]. However, this complexity might be advantageous in terms of anatomical preservation. Because StarGAN has to optimize for both CBCT and MRI, it also learns more about anatomical representations present in both CBCT and MRI, thus enhancing the model’s ability to preserve anatomy in the generated images. In our study, we only used three image domains, with two of them being quite similar (CBCT and pCT) compared with the third (MRI). Therefore, the StarGAN model tends to optimize itself more toward these two types. This is evident from the fact that the lowest MAE in transfer training occurs in the early epochs (epoch 9 out of 250), while the later epochs focus more on optimizing for CBCT and pCT. For future studies, it is necessary to assess whether incorporating more MRI sequences to increase the number of MRI images for model optimization or to using more MRI data compared with the other domains leads to better optimization of the StarGAN model toward MRI images.

### Limitations and future work

A significant limitation of this study was the availability of MRI data. The MRI sequences required to generate sCT—with an FOV that covers the full body in the axial plane—are not routine in our institution’s radiotherapy workflow, so we had limited training data and potential robustness issues. The shortage of MRI data for testing also limited the depth of our observations. Future research should aim to incorporate more MRI data, but researchers must consider these challenges to ensure their findings are valid. Another limitation is the data usage bias in StarGAN, which uses both MRI and CBCT images, consuming more data compared with CycleGAN. While we viewed this as an advantage in this study, this aspect warrants further investigation in future research. Moreover, this study applies StarGAN in a 2D manner. Future research could explore a 3D implementation, which might yield improved results. Additionally, this study is retrospective, relying on previously treated plans recalculated on sCT without directly evaluating the contouring and planning processes on the sCT. Future studies should assess the feasibility of integrating sCT into the full clinical workflow, including contouring and treatment planning.

## Conclusion

In summary, StarGAN can generate sCT from CBCT and MRI with comparable quality as widely used CycleGAN with better anatomical preservation. The models can enhance the HU values in CBCT and produce high-quality sCT from MRI, showing significant promise for enabling MRI simulation and ART using CBCT or MRI, potentially streamlining radiotherapy workflows.

## Supplementary Information

Additional file 1.

## Data Availability

No datasets were generated or analysed during the current study.

## Abbreviations

ART: Adaptive Radiation Therapy
CBCT: Cone Beam Computed Tomography
CT: Computed Tomography
DD: Dose Differences
DSC: Dice Similarity Coefficient
DVH: Dose-Volume Histogram
FOV: Field of View
GAN: Generative Adversarial Network
GPU: Graphics Processing Unit
HU: Hounsfield Units
IMRT: Intensity-Modulated Radiation Therapy
MAE: Mean Absolute Error
MRI: Magnetic Resonance Imaging
pCT: Planning Computed Tomography
PSNR: Peak Signal-to-Noise Ratio
PTV: Planning Target Volume
ROI: Region of Interest
sCT: Synthetic Computed Tomography
SSIM: Structural Similarity Index
TPS: Treatment Planning System
VMAT: Volumetric Modulated Arc Therapy
