# Supplementary material for: Synthetic CT generation from CBCT and MRI using StarGAN in the Pelvic Region
Source: Radiat Oncol. 2025 Feb 4;20:18. doi: 10.1186/s13014-025-02590-2 (PMC11796232; doi:10.1186/s13014-025-02590-2)
Supplement: Supplementary file 1 — Additional file 1. [file 13014_2025_2590_MOESM1_ESM.docx]

**Table S1:** Details of patients in this study used across training, testing, and validation phases for evaluating CycleGAN and StarGAN models. (“/” indicates that the data is available)

| **Sex** | CBCT equipment | **Diag** | **CT** | **CBCT** | **MRI** | **CBCT CycleGAN** | **MRI CycleGAN** | **StarGAN** |
| --- | --- | --- | --- | --- | --- | --- | --- | --- |
| M | Varian iX | CA Prostate | / | / | / | test | test | test |
| M | Varian Edge | CA Prostate | / | / | / | test | test | test |
| M | Varian Edge | CA Prostate | / | / | / | test | test | test |
| M | Varian Edge | CA Prostate | / | / | / | test | test | test |
| M |  | CA Bladder | / |  | / |  | test | test |
| M | Varian Truebeam | CA Prostate | / | / |  | test |  | test |
| F | Varian iX | CA Rectum | / | / | / | train | train | train |
| M | Varian Truebeam | CA Rectum | / | / | / | train | train | train |
| F | Varian Truebeam | CA Bladder | / | / | / | train | train | train |
| F | Varian Truebeam | CA Cervix | / | / | / | train | train | train |
| F | Varian Truebeam | CA Rectum | / | / | / | train | train | train |
| M | Varian Truebeam | CA Rectum | / | / | / | train | train | train |
| F | Varian Truebeam | CA Cervix | / | / | / | train | train | train |
| F | Varian Edge | CA Rectum | / | / | / |  | train | train |
| M | Varian iX | CA Cervix | / | / | / |  | train | train |
| M |  | CA Prostate | / |  | / |  | train | train |
| M |  | CA Prostate | / |  | / |  | train | train |
| M |  | CA Prostate | / |  | / |  | train | train |
| M |  | CA Prostate | / |  | / |  | train | train |
| F |  | CA Rectum | / |  | / |  | train | train |
| M |  | CA Rectum | / |  | / |  | train | train |
| F |  | CA Rectum | / |  | / |  | train | train |
| F |  | CA Cervix | / |  | / |  | train | train |
| F |  | CA Cervix | / |  | / |  | train | train |
| F |  | CA Cervix | / |  | / |  | train | train |
| F |  | CA Cervix | / |  | / |  | train | train |
| F |  | CA Cervix | / |  | / |  | train | train |
| M |  | CA Prostate | / |  | / |  | train | train |
| M |  | CA Prostate | / |  | / |  | train | train |
| M | Varian Truebeam | CA Prostate | / | / |  | train |  | train |
| M | Varian Truebeam | CA Prostate | / | / |  | train |  | train |
| M | Varian Truebeam | CA Prostate | / | / |  | train |  | train |
| M | Varian Truebeam | CA Prostate | / | / |  | train |  | train |
| M | Varian Truebeam | CA Rectum | / | / |  | train |  | train |
| M | Varian Truebeam | CA Pyriform | / | / |  | train |  | train |
| F | Varian Truebeam | CA Cervix | / | / |  | train |  | train |
| M | Varian Truebeam | CA Prostate | / | / |  | train |  | train |
| F | Varian Truebeam | CA Pancreas | / | / |  | train |  | train |
| M | Varian Truebeam | CA Prostate | / | / |  | train |  | train |
| M | Varian Truebeam | CA Prostate | / | / |  | train |  | train |
| M | Varian Truebeam | CA Prostate | / | / |  | train |  | train |
| M | Varian Truebeam | CA Prostate | / | / |  | train |  | train |
| M | Varian Truebeam | CA Prostate | / | / |  | train |  | train |
| M | Varian Truebeam | CA Prostate | / | / |  | train |  | train |
| M | Varian Truebeam | CA Prostate | / | / |  | train |  | train |
| F | Varian Truebeam | CA Endometrium | / | / |  |  |  | train |
| M | Varian Truebeam | CA Prostate | / | / |  |  |  | train |
| M | Varian Edge | CA Prostate | / | / |  |  |  | train |
| M | Varian Truebeam | CA Rectum | / | / | / | validation | validation | validation |
| M | Varian iX | CA Prostate | / | / | / | validation | validation | validation |
| F | Varian Truebeam | CA Rectum | / | / | / | validation | validation | validation |
| M | Varian iX | CA Rectum | / | / | / | validation | validation | validation |
| M | Varian iX | CA Rectum | / | / | / | validation | validation | validation |

Table S2: Mean SSIM ± SD and PSNR ± SD of CBCT, sCT from CBCT and MRI by StarGAN and CycleGAN using pCT as a reference.

| **Base image** | **Model** | **SSIM** | | **PSNR (dB)** | |
| --- | --- | --- | --- | --- | --- |
|  |  | **Mean ± SD** | **p-value** | **Mean ± SD** | **p-value** |
| **CBCT** | **CBCT** | 0.86 ± 0.03 | - | 29.36 ± 0.77 | - |
|  | **CycleGAN** | 0.88 ± 0.02 | 0.1797 | 25.73 ± 0.69 | 0.0625 |
|  | **StarGAN** | 0.88 ± 0.02 |  | 29.18 ± 1.07 |  |
| **MRI** | **CycleGAN** | 0.85 ± 0.03 | 0.0656 | 24.74 ± 0.89 | 0.0625 |
|  | **StarGAN** | 0.82 ± 0.02 |  | 23.81 ± 0.89 |  |

Table S3: Mean Dose difference (DD) ± SD in test dataset of CBCT and sCT from CBCT and MRI by StarGAN and CycleGAN using pCT as a reference.

|  | **Base image** | **Model** | **%DD ± Range** | **p-value** | **Criteria** [30,31] |
| --- | --- | --- | --- | --- | --- |
| **PTV D95%** | **CBCT** | **CBCT** | 1.15 ± 0.96 | **-** | <2% |
|  |  | **CycleGAN** | -1.88 ± 0.35 | 0.0625 |  |
|  |  | **StarGAN** | 0.38 ± 0.48 |  |  |
|  | **MRI** | **CycleGAN** | -1.02 ± 2.44 | 0.6250 |  |
|  |  | **StarGAN** | 1.27 ± 0.23 |  |  |
| **PTV D2%** | **CBCT** | **CBCT** | 1.19 ± 0.91 | - |  |
|  |  | **CycleGAN** | -1.49 ± 0.46 | 0.0625 |  |
|  |  | **StarGAN** | 0.63 ± 0.3 |  |  |
|  | **MRI** | **CycleGAN** | 0.45 ± 0.87 | 0.0625 |  |
|  |  | **StarGAN** | 1.75 ± 0.44 |  |  |
| **PTV Dmean** | **CBCT** | **CBCT** | 1.12 ± 0.93 | - |  |
|  |  | **CycleGAN** | -1.61 ± 0.45 | 0.0625 |  |
|  |  | **StarGAN** | 0.53 ± 0.34 |  |  |
|  | **MRI** | **CycleGAN** | 0.18 ± 0.9 | 0.0625 |  |
|  |  | **StarGAN** | 1.47 ± 0.41 |  |  |
| **Body D2%** | **CBCT** | **CBCT** | 1.28 ± 1.34 | - |  |
|  |  | **CycleGAN** | -3.38 ± 0.63 | 0.0625 |  |
|  |  | **StarGAN** | 0.21 ± 0.76 |  |  |
|  | **MRI** | **CycleGAN** | 0.6 ± 1.69 | 0.6250 |  |
|  |  | **StarGAN** | 1.94 ± 1.34 |  |  |
| **Body Dmean** | **CBCT** | **CBCT** | 0.83 ± 1.15 | - |  |
|  |  | **CycleGAN** | -2.91 ± 0.73 | 0.0625 |  |
|  |  | **StarGAN** | 0.01 ± 0.6 |  |  |
|  | **MRI** | **CycleGAN** | -0.01 ± 1.58 | 0.8125 |  |
|  |  | **StarGAN** | 1.76 ± 1.07 |  |  |

**Table S4:** Mean percentage gamma passing rate (%GPR) ± SD using global normalization in test dataset at low dose cutoff of 50%.

| **Gamma Criteria** | **Low dose cutoff** | **Base image** | **Model** | **%GPR ± SD** | **p-value** |
| --- | --- | --- | --- | --- | --- |
| **2%/2 mm** | **50%** | **CBCT** | **CBCT** | 99.16 ± 1.5 | - |
|  |  |  | **CycleGAN** | 97.65 ± 3.53 | 0.0625 |
|  |  |  | **StarGAN** | 100.00 ± 0.00 | 0.1088 |
|  |  | **MRI** | **CycleGAN** | 99.37 ± 1.38 | 0.0625 |
|  |  |  | **StarGAN** | 92.41 ± 4.73 | 0.0710 |
